# *Toxoplasma gondii*-induced adverse pregnancy outcomes: insight into the inhibitory role of Trem2 on TLR4/TRAF6/JNK signaling pathway

**DOI:** 10.1186/s13071-025-07000-w

**Published:** 2025-10-06

**Authors:** Yining Cao, Feifei Fu, Fei Ju, Chenyu Wu, Tiankun Yao, Mei Yang, Baolan Sun, Jinling Chen

**Affiliations:** 1 Department of Pathogen Biology, Medical School of Nantong University, 19 Qixiu Road, Nantong, 226001 Jiangsu People’s Republic of China; 2 Department of Laboratory, Affiliated Hospital of Nantong University, 20 Xisi Road, Nantong, 226001 Jiangsu People’s Republic of China; 3 Research Center of Clinical Medicine, Affiliated Hospital of Nantong University, Nantong, 226001 Jiangsu People’s Republic of China

**Keywords:** *Toxoplasma gondii*, Adverse pregnancy outcomes, Triggering receptor expressed on myeloid cells 2, Macrophages, TLR4/TRAF6/JNK signaling pathway

## Abstract

**Background:**

Decidual macrophages (dMφs) are not only essential for maintaining normal pregnancy but also serve as crucial immune defenders against infections, including *Toxoplasma gondii*. Triggering receptor expressed on myeloid cells 2 (Trem2), as a critical immunoregulatory receptor on dMφs, can counteract inflammation and defend against pathogen infection. However, the mechanisms underlying the Trem2 downstream pathways during *T. gondii* infection—particularly their impact on adverse pregnancy outcomes (APOs)—remain elusive.

**Methods:**

The interaction between Trem2 and Toll-like receptor 4 (TLR4) was initially predicted through molecular docking models and subsequently confirmed by co-immunoprecipitation, using both animal models and cellular systems to examine the impact of *Trem2* knockout, overexpression, and TLR4-blocking antibody treatment on downstream signaling molecules as well as cytokine production.

**Results:**

The interaction between Trem2 and TLR4 was validated. Trem2 downregulation during *T. gondii* infection coincided with increased TLR4, tumor necrosis factor (TNF) receptor-associated factor 6 (TRAF6), and c-Jun N-terminal kinase (JNK) activation, while *Trem2* knockout further enhanced TLR4/TRAF6/JNK signaling in mice and macrophages. Conversely, *Trem2* overexpression suppressed this signaling cascade and reversed *T. gondii*-induced activation. Treatment with a TLR4-blocking antibody inhibited TRAF6 and P-JNK activation in macrophages but did not affect Trem2 expression. Additionally, *Trem2*-deficient bone marrow-derived macrophages (BMDMs) exhibited elevated transcription of *TNF-α* and interferon-γ (*IFN-γ*) upon *T. gondii* antigen stimulation.

**Conclusions:**

*Trem2* deficiency in pregnant mice promotes the TLR4/TRAF6/JNK signaling cascade following *T. gondii* infection. This study demonstrates that Trem2 acts as a pregnancy-specific inhibitor of TLR4/TRAF6/JNK signaling, providing novel mechanistic insights into *T. gondii*-induced APOs.

**Graphical Abstract:**

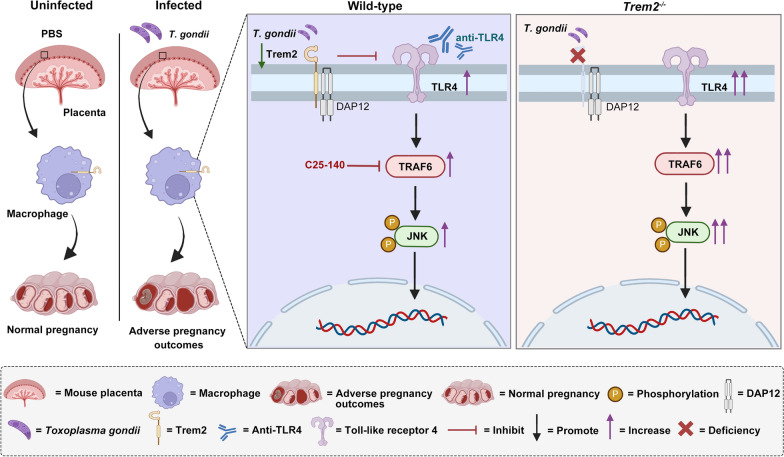

**Supplementary Information:**

The online version contains supplementary material available at 10.1186/s13071-025-07000-w.

## Background

*Toxoplasma gondii*, as a zoonotic apicomplexan protozoan, poses a significant risk for human health [1]. It is estimated that *T. gondii* infects up to one-third of the global human population [2]. It is also a major pathogen responsible for infection-related adverse pregnancy outcomes (APOs) in both humans and livestock [3]. Primary infection acquired during pregnancy can result in severe damage to the fetus via the maternal–fetal interface, including miscarriage, preterm labor, and stillbirth [4]. The maternal–fetal interface comprises a diverse array of decidual immunocytes, including natural killer (NK) cells, macrophages, T cells, and dendritic cells (DCs), which cooperate to sustain immune homeostasis, support maternal–fetal tolerance, and maintain the defense competence against pathogens. Hence, an imbalance in immune tolerance of the maternal–fetal interface is recognized as a contributing factor to APOs [5]. *Toxoplasma gondii* can infect nearly all nucleated cells, with macrophages and DCs preferentially targeted for parasite transmission [4]. Furthermore, increasing evidence has highlighted the critical impact of decidual macrophages (dMφs) on maintaining immune tolerance of the maternal–fetal interface [6]. Comprising 20% of the maternal–fetal interface, dMφs engage in tissue remodeling of the placenta by secreting matrix metalloproteinases (MMPs) and eliminating foreign pathogens via phagocytosis [7, 8]. Additionally, dMφs release inflammatory cytokines, which can disrupt immune tolerance and induce the development of pathological pregnancy conditions [9]. Evidence indicates that *T. gondii* infection induces quantitative and qualitative changes in dMφ populations [10]. In pregnant mice with knockout of galectin-9 (GAI-9) and placental growth factor (*Pgf*), *T. gondii* infection elicited a more pronounced impairment of dMφ function, leading to more severe APOs [9, 11]. These molecular markers, however, are not selectively expressed in macrophages. The precise mechanisms by which macrophage-specific functional molecules contribute to sustaining immune homeostasis during pregnancy remain unclear.

Triggering receptor expressed on myeloid cells 2 (Trem2), as a key immunoregulatory receptor, was initially identified in macrophages and DCs derived from monocytes. It is tightly linked with the pathogenic progression of Alzheimer’s disease [12], lung cancer [13], and nonalcoholic steatohepatitis [14]. Nevertheless, recent studies have demonstrated that Trem2 can also be expressed in dMφs, and the number of *Trem2*^*+*^ macrophages is decreased in patients with preeclampsia (PE) [15, 16]. In addition, Trem2 functions as a pathogen phagocytic receptor in various infectious diseases [17], including its role in regulating *Salmonella enterica* serovar Typhimurium infection [18]. Reactive oxygen species (ROS) released from macrophages, a major key defender against *Salmonella* infection, require the coordinated signaling of DAP12 and Trem2 [18]. Trem2 may play a protective role against *T. gondii-*induced APOs by governing the dMφ function through the spleen tyrosine kinase/phosphoinositide 3-kinase (SYK/PI3K) signaling pathway [19]. Importantly, Trem2 exerts an anti-inflammatory effect in Parkinson’s disease through the Toll-like receptor 4 (TLR4)-mediated myeloid differentiation primary response protein 88 (MyD88)/nuclear factor-kB (NF-κB) signal cascade [20]. TLR signaling activates NF-κB and promotes the inflammatory signaling pathway through the recruitment of adaptor proteins including MyD88 as well as tumor necrosis factor (TNF) receptor-associated factor 6 (TRAF6) [21, 22]. TRAF6, as a pivotal adaptor molecule in the TLR-related signaling pathway, mediates downstream activation of the TAK1-binding protein 2 (TAB2)/TAK1 complex to trigger the mitogen-activated protein kinase (MAPK) cascade, thereby regulating inflammation reaction and cytokine production [23, 24]. More importantly, TLR4, but not TLR2, was found to be elevated in interstitial trophoblasts from patients with PE, indicating that TLR4-mediated innate immune activation may act as a contributing factor to APOs [25]. However, it remains elusive whether or how Trem2 regulates the TLR4/TRAF6/JNK axis in *T. gondii*-triggered APOs.

Here, we found that *Trem2* deficiency amplified TLR4-TRAF6-JNK signaling cascade activation in pregnant murine models challenged with *T. gondii*. We identified the interaction of Trem2 with TLR4. Mechanistic investigations revealed that Trem2 inhibited the TLR4/TRAF6/JNK axis and suppressed the transcription of *TNF-α* and interferon-γ (*IFN-γ*). Therefore, therapeutic strategies aimed at upregulating Trem2 expression to inhibit the TLR4/TRAF6/JNK signaling pathway may represent a promising approach for treating APOs, especially those triggered by *T. gondii*.

## Methods

### Experimental animals

*Trem2*^*−/−*^ mice, kept on the C57BL/6 genetic background, were obtained from Gempharmatech Co., Ltd. (Nanjing, Jiangsu, China). Wild type (WT) and *Trem2*^*−/−*^ mice were bred and kept under specific-pathogen-free conditions at the Laboratory Animal Center of Nantong University. Adult female mice (8 weeks old) and male mice (10 weeks old) were paired at a 2:1 ratio overnight for mating. The female mouse was inspected in the morning for the presence of a vaginal plug, with this time point designated as gestational day 0.5 (G0.5) [26]. WT and *Trem2*^*−/−*^ pregnant mice were divided into phosphate-buffered saline (PBS) control and *T. gondii* (RH strain) infection groups. At G8.5, mice were intraperitoneally injected with either 300 tachyzoites in 200 μl PBS (infection group) or the same volume of PBS alone (control group) [27]. Placental and fetal tissues were collected after all pregnant mice were euthanized at G17.5 using carbon dioxide (CO_2_) inhalation asphyxiation.

### Preparation of antigens derived from *T. gondii*

*Toxoplasma gondii* antigens (*Tg*Ag) were prepared according to previously established protocols [28]. Briefly, 1 × 10^8^ tachyzoites of *T. gondii* were cultured in 10 ml of serum-free RPMI 1640 (Thermo Fisher Scientific, Waltham, MA, USA) medium under mild agitation for 3 h in a 37 °C atmosphere. After collection, the culture supernatant was first centrifuged and then concentrated using an Amicon^®^ Ultra-15 centrifugal filter (10 kDa MWCO; Merck Millipore, Darmstadt, Germany). Endotoxins were removed using an AffinityPakDetoxi-Gel kit (Thermo Fisher Scientific), and the *Tg*Ag-containing fluid was filtered via a 0.22-µm membrane. *Tg*Ag protein quantification was performed via Bradford colorimetric analysis (Thermo Fisher Scientific).

### Cell culture and treatment

RAW 264.7 cells, leukemia cells of mouse mononuclear macrophage, were obtained from the Chinese Academy of Sciences Cell Bank and cultured in complete Dulbecco’s modified Eagle medium (DMEM; Thermo Fisher Scientific) containing 10% fetal bovine serum (FBS; ExCell, Suzhou, Jiangsu, China) and 1% penicillin–streptomycin solution in 5% CO_2_, 37 °C atmosphere. RAW 264.7 cells were exposed to *Tg*Ag (5 μg/ml) for 24 h to mimic *T. gondii* (RH strain) infection in vitro. To inhibit TRAF6 expression, C25-140 (20 μM; Med Chem Express, NJ, USA) was administered to RAW 264.7 cells for 2 h, which were then stimulated with/without *Tg*Ag (5 μg/ml) for 24 h. To investigate the Trem2-related downstream signaling pathway, RAW 264.7 cells were primed with either TLR4/MD-2 complex antibody (20 μg/ml; 16-9924-81, Thermo Fisher Scientific) or isotype-matched IgG (20 μg/ml; 16-4321-82, Thermo Fisher Scientific) for 30 min and then stimulated with/without *Tg*Ag (5 μg/ml) for 24 h.

For *Trem2* overexpression in RAW 264.7 cells, the *Trem2-DAP12* chimeras were constructed following established experimental protocols [29]. PcSLenti-EF1-EGFP-F2A-Puro-CMV-Igk-leader-*Trem2* (19-177aa)-Tyrobp (28-144a)-WPRE and pcSLenti-EF1-EGFP-F2A-Puro-CMV-MCS-WPRE, obtained from Obio Technology (Shanghai, China), were transfected into RAW 264.7 cells at a multiplicity of infection (MOI) of 120 for 72 h per the manufacturer’s instructions. Then, puromycin (Beyotime, Shanghai, China) was added to the medium to a final concentration of 2 μg/ml for the selection of stably transduced cell clones. Finally, the RAW 264.7 cell line with stable overexpression of GFP-*Trem2* (RAW 264.7-*Trem2* cells) was obtained.

The sequences of si-*Trem2* and negative control (si-NC), obtained from GenePharma (Shanghai, China), are provided as follows (5′ to 3′): si-*Trem2* sense: GGACCCUCUAGAUGACCAATT, si-*Trem2* antisense: UUGGUCAUCUAGAGGUCCTT; negative control: sense: UUCUCCGAACGUGUCACGUTT, antisense: ACGUGACACGUUCGGAGAATT. Then, si-*Trem2* (5 μl) and si-NC (5 μl) were respectively mixed with serum-free DMEM medium (200 μl) and vortexed for 30 s. The serum-free DMEM medium (200 μl) was then combined with 5 μl INTERFERin transfection reagent (PolyPlus, Strasbourg, France). RAW 264.7 cells (2 × 10^5^/well) were planted and cultured overnight in six-well plates. Upon reaching 40–50% confluence, RAW 264.7 cells were incubated with the mixture of the two reagents at room temperature (RT) for 15 min. The culture medium was removed and replaced with fresh complete DMEM at 4 h post-transfection.

### Immunofluorescence and image processing

RAW 264.7 cells were cultured on a coverslip (Biosharp, Hefei, Anhui, China) to 40–50% confluence and treated with/without *Tg*Ag (5 μg/ml) for 24 h. Following fixation with 4% paraformaldehyde (PFA; Biosharp) and permeabilization with 0.1% Triton X-100, RAW 264.7 cells were blocked by 5% bovine serum albumin/Tris-buffered saline (BSA-TBS) for at least 30 min and incubated with primary antibody for 1 h at RT. All reagents used in the subsequent steps were obtained from the TSA reagent kit (Absin, Shanghai, China). RAW 264.7 cells were incubated with anti-rabbit secondary antibody (10 min, RT), washed with 1× TBST 3 times, and then incubated with different fluorescent dyes (10 min, RT), culminating in nuclear counterstaining with DAPI (1:100 dilution in double-distilled [dd]H_2_O, 5 min). The coverslip was mounted in an anti-fade mounting medium, and a photograph was obtained with an Olympus FV3000 confocal microscope (Olympus, Tokyo, Japan). Dilutions of primary antibodies were as follows: anti-TRAF6 antibody (1:250, rabbit monoclonal, ab40675, Abcam, Cambridge, MA, USA), anti-TLR4 antibody (1:100, mouse monoclonal, 66350-1-Ig, Proteintech, Rosemont, IL, USA), and anti-Phospho-SAPK/JNK antibody (Thr183/Tyr185; 1:50, rabbit monoclonal, 4668, Cell Signaling Technology, Danvers, MA, USA).

### Western blotting

Either mouse placentas or RAW 264.7 cells were resuspended in radioimmunoprecipitation assay (RIPA) lysis buffer (1 M Tris–HCl, 0.5 M ethylenediaminetetraacetic acid [EDTA], 1% TritonX-100) with phenylmethylsulfonyl fluoride (PMSF; Beyotime) and phosphatase inhibitor cocktail (Sangon, Shanghai, China) on ice for 20 min, and subjected to sonication for 10 s. The protein lysates with loading buffer were boiled at 100 °C for 10 min, loaded into 10% or 8% sodium dodecyl sulphate–polyacrylamide gel electrophoresis (SDS–PAGE) with equal amounts of protein, and blotted onto a 0.45 μm polyvinylidene fluoride (PVDF; Merck Millipore) membrane. After blockage with 5% fat-free milk (w/v) in 100 ml 1× TBST at RT for 1 h, the PVDF membrane was incubated with primary antibody overnight at 4 ℃. Subsequent immunodetection was performed using secondary antibodies, and signal development was achieved through the enhanced chemiluminescence (ECL, MA0186-1; Meilunbio, Liaoning, China).

The following antibodies were utilized in the experiments: anti-TRAF6 antibody (1:2000, rabbit monoclonal, ab40675, Abcam), anti-TLR4 antibody (1:1000, mouse monoclonal, 66350-1-Ig, Proteintech), anti-Phospho-SAPK/JNK antibody (Thr183/Tyr185; 1:1000, rabbit monoclonal, 4668, Cell Signaling Technology), anti-GAPDH (1:50,000, mouse monoclonal, 60004-1-Ig, Proteintech), anti-mouse HRP-IgG (1:5000, SA00001-1, Proteintech), anti-rabbit HRP-IgG (1:5000, SA00001-2, Proteintech), anti-Trem2 antibody (1:3000, sheep monoclonal, AF1729, R&D Systems), and anti-sheep HRP-IgG (1:5000; sc2473, Santa Cruz Biotechnology, Santa Cruz, CA, USA).

### Immunoprecipitation

RAW 264.7 cells, exposed to *Tg*Ag for 24 h, were washed and lysed on ice for 45 min with an immunoprecipitation binding buffer (20 mM Tris [pH 7.5], 150 mM NaCl, 1% Triton X-100; P0013J, Beyotime), followed by sonication at 300 W for 5 s. 10% of the whole cell lysate was provided as an input control. Immunoprecipitation was performed using Trem2 antibody (2 μg; rabbit monoclonal, ab305103; Abcam) and isotype control (2 μg; rabbit [DA1E] mAb IgG XP Isotype Control^®^, 3900; Cell Signaling Technology). Following overnight incubation, Protein A/G agarose beads (sc2003; Santa Cruz Biotechnology) were incorporated into the mixture and then rotated at 4 ℃ for 2 h. Finally, the protein sample was separated by SDS–PAGE, immunoblotted with the corresponding antibody, and developed with ECL.

### Isolation and differentiation of bone marrow-derived macrophages

Isolation and differentiation of bone marrow-derived macrophages (BMDMs) from either C57BL/6 or *Trem2*^*−/−*^ mice (8–10 weeks old) were conducted according to the following protocol [30]. Femurs and tibiae were harvested and subsequently immersed in complete DMEM media containing 10% FBS and 1% penicillin–streptomycin solution. Bone marrow was extracted through multiple flushes using complete DMEM media to collect marrow cells. To eliminate erythrocyte contamination, the cell suspension was treated with ice-cold erythrocyte lysis buffer (Sangon) at RT for 15 min and then centrifuged at 300×g at 4 ℃ for 15 min.

Primary cell cultures were established by maintaining isolated cells in medium enriched with macrophage colony-stimulating factor (40 ng/ml; M-CSF, PeproTech, Rocky Hill, NJ, USA). Partial medium renewal (50% volume replacement) was performed on culture days 3 and 6. Following 7 days of differentiation under M-CSF stimulation, fully differentiated BMDMs were harvested for experimental applications.

### Molecular docking

The protein crystal structures of TLR4 and Trem2 were retrieved using the Research Collaboratory for Structural Bioinformatics (RCSB) Protein Data Bank (http://www.pdb.org/), with molecular docking models established via GRAMM Web (https://gramm.compbio.ku.edu/). The highest-scoring model structures were then selected for image generation in the PyMOL molecular graphics system (Schrodinger LLC, NY, UK).

### Quantitative real-time PCR

RNA, isolated from either *Tg*Ag-treated or untreated BMDMs of WT mice and *Trem2*^*−/−*^ mice, was performed with TRIzol reagent (Thermo Fisher Scientific). Then, complementary DNA (cDNA) synthesis was conducted with the specified reverse transcription kit (Thermo Fisher Scientific). The analysis of quantitative polymerase chain reaction (qPCR) was executed with SYBR Premix Ex Taq II (Tli RNaseH Plus) kit (Takara, Kyoto, Japan) and then monitored through the Applied Biosystems detection platform (Carlsbad, CA, USA). All primer sequences are listed in Additional file 1: Table S1.

### Statistical analysis

Data were expressed as mean ± standard deviation (SD). Statistical analysis was conducted with GraphPad Prism 9.0 (GraphPad Software, La Jolla, CA, USA). Continuous variables were compared between the two groups with an unpaired two-tailed Student *t*-test. One-way analysis of variance (ANOVA) with Tukey’s multiple comparisons test was employed to compare three or more groups. When analyzing datasets with three or more groups across two variables, two-way ANOVA with Sidak’s multiple comparisons test was conducted to evaluate the significance of the difference (*P* values less than 0.05). Specific sample sizes (*n*-values) for each analysis were shown in the corresponding figure legends.

## Results

### The absence of *Trem2* further promoted *T. gondii*-triggered upregulation of TLR4, TRAF6, and P-JNK in mouse placentas

**Fig. 1:**
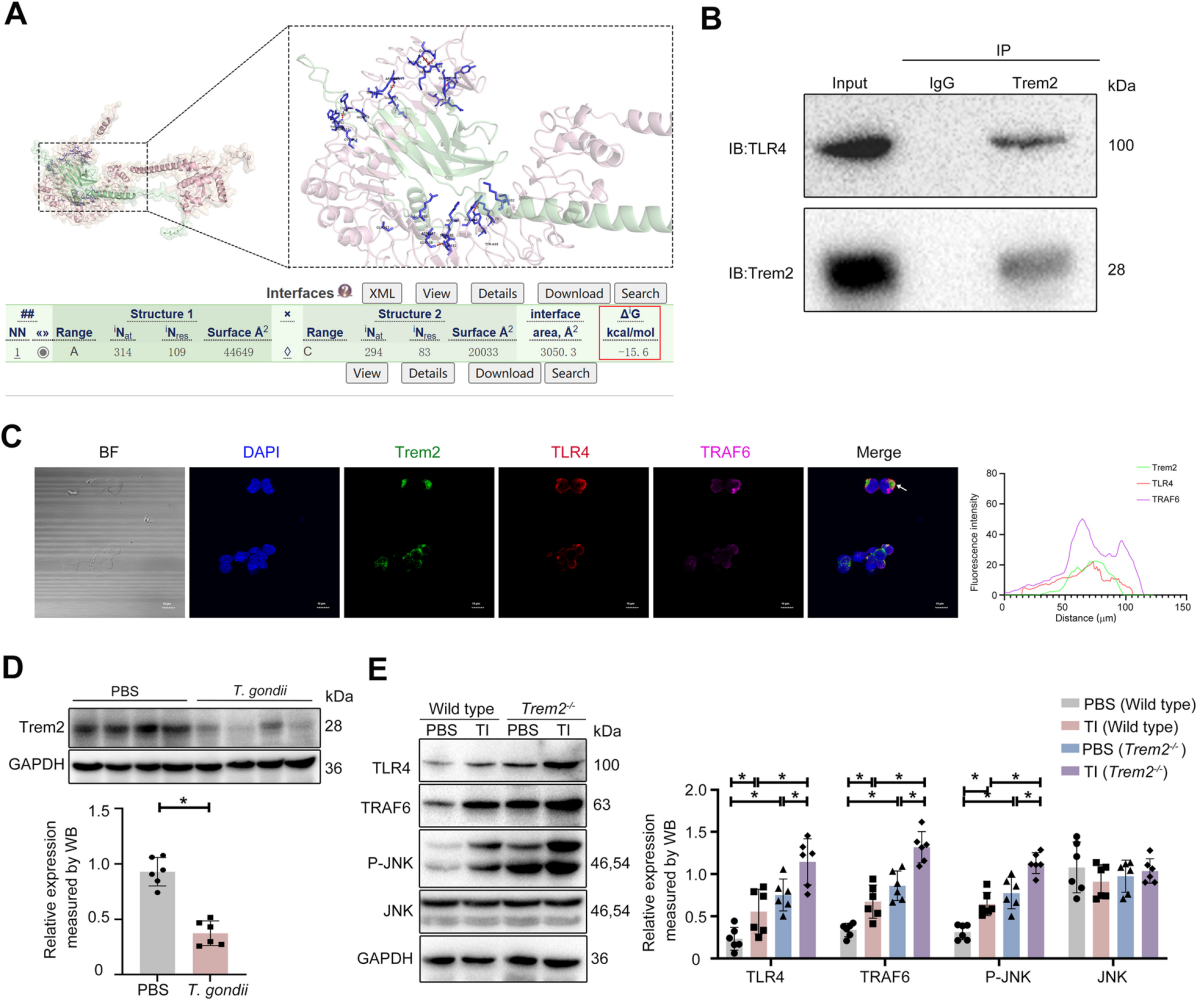
The absence of *Trem2* further promoted the *T. gondii*-triggered upregulation of TLR4, TRAF6, and P-JNK in mouse placentas. **A** The docking models of TLR4 and Trem2 were predicted using the GRAMM docking software and visualized with PyMol. The calculated binding energy between TLR4 (green) and Trem2 (pink) was −15 kcal/mol. **B** Endogenous immunoprecipitation was conducted to assess the interaction between Trem2 and TLR4. RAW 264.7 cell lysate was immunoprecipitated with Trem2 antibody. Expression of Trem2 and TLR4 was assayed by western blotting. **C** Multiplex immunofluorescence analysis was conducted to examine the colocalization of TLR4, Trem2, and TRAF6. RAW 264.7 cells were incubated with Trem2 antibody (green), TLR4 antibody (red), TRAF6 antibody (magenta), and DAPI dye (blue). Fluorescence intensity and co-localization of the three proteins were analyzed using ImageJ software. Scale bar: 10 μm. **D** Expression of Trem2 in the placentas from WT pregnant mice challenged with/without *T. gondii*, along with corresponding quantitative analysis. Placental tissue was extracted from WT pregnant mice on G17.5, and western blotting was employed to assay Trem2 expression (*n* = 6). **E** Expression of TLR4, JNK, P-JNK, and TRAF6 in placental tissues from *Trem2*^*−/−*^ and WT pregnant mice challenged with/without *T. gondii*, along with corresponding quantitative analysis. Placental tissue was extracted from WT and *Trem2*^*−/−*^ pregnant mice on G17.5. Western blotting was employed to assay the level of TLR4, JNK, P-JNK, and TRAF6 (*n* = 6). WT: Wild type, TI: *T. gondii* infection. Significant changes were determined with a two-tailed unpaired Student *t*-test (**D**) and two-way ANOVA with Sidak’s multiple comparisons test (**E**) at *P* < 0.05. *P < 0.05

Our previous research indicated that Trem2 serves as a critical protective factor during *T. gondii* infection, and its deficiency exacerbates APOs [19]. This study was conducted to explore Trem2 downstream signaling pathways to further investigate the underlying mechanisms. TLR4 is known to mediate the inflammation response to *T. gondii* infection via parasite-derived heat shock protein 70 (HSP70) [31]. Studies have shown that Trem2 activation suppresses TLR4-dependent inflammatory responses by modulating its signaling pathway [32]. TRAF6, acting as an E3 ubiquitin ligase, can be recruited to the TLR4-activated signaling complex and subsequently facilitates the activation of downstream signaling molecules, including JNK, ERK1/2 (extracellular regulated protein kinases 1/2), and p38 [21]. Owing to these observations, we supposed that *T. gondii* infection during pregnancy in mice might inhibit Trem2 expression and subsequently enhance the TLR4-dependent signaling pathway. We first employed the GRAMM Web Server database to predict potential binding sites between Trem2 and TLR4, with a calculated binding energy (−15 kcal/mol), indicating a direct interaction, and visualized the predicted complex using PyMOL (Fig. 1A). These results suggested direct physical interaction between TLR4 and Trem2. Subsequent endogenous co-immunoprecipitation assays in RAW 264.7 cells confirmed the association of Trem2 with TLR4 (Fig. 1B). Multiplex immunofluorescence analysis further revealed that Trem2 can co-localize with TLR4. Notably, the colocalization of TLR4, Trem2, and TRAF6 was also observed, implying potential functional coordination among these molecules (Fig. 1C). Subsequently, we investigated the regulatory mechanism of Trem2 and its downstream signaling molecules to further explore the pathogenesis of APOs induced by *T. gondii* infection. TLR4-related signaling molecules were assayed in the placental tissues of mice challenged with *T. gondii.* Immunoblotting analysis indicated that *T. gondii* infection markedly reduced Trem2 expression (Fig. 1D), but upregulated TLR4, along with its downstream signaling molecules, including P-JNK and TRAF6 (Fig. 1E). Intriguingly, in comparison with WT mice, *Trem2*^*−/−*^ mice exhibited the enhanced expression of TLR4 and its downstream signaling molecules in placental tissues. Furthermore, *T. gondii* infection further amplified the expression of TLR4, P-JNK, and TRAF6 in the placentas from *Trem2*^*−/−*^ mice (Fig. 1E). Taken together, these data underscored that *Trem2* deficiency exacerbated APOs in *T. gondii*-challenged mice, potentially through the upregulation of TLR4-mediated signaling pathways.

### *Toxoplasma gondii* antigens suppressed Trem2 expression to activate downstream TLR4-dependent signaling

**Fig. 2:**
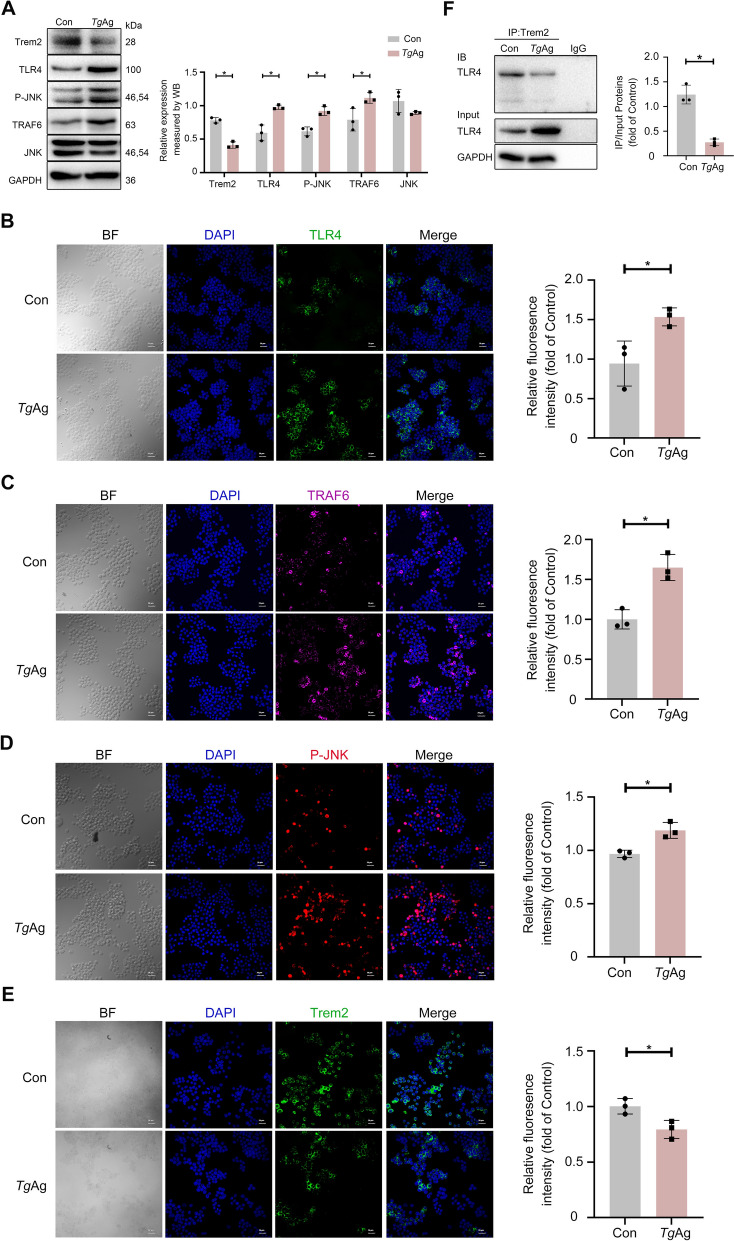
*Toxoplasma gondii* antigens suppressed Trem2 expression but activated the downstream TLR4 signaling pathway in RAW 264.7 cells. **A** Immunoblot analysis of RAW 264.7 cells stimulated with/without *Tg*Ag. Western blotting was conducted to measure the expression of TLR4, P-JNK, JNK, TRAF6, and Trem2 in RAW 264.7 cells that were treated with/without *Tg*Ag (5 µg/ml) for 24 h (*n* = 3). **B**–**E** Representative immunofluorescence photograph and quantitative analysis of the expression of TLR4, TRAF6, and P-JNK in RAW 264.7 cells stimulated with/without *Tg*Ag. Immunofluorescence was used to assess the fluorescence intensity of TLR4 (green), TRAF6 (magenta), P-JNK (red), and Trem2 (green) in RAW 264.7 cells stimulated with/without *Tg*Ag (5 μg/ml) for 24 h. Scale bar: 20 μm. Fluorescence intensity was quantified by using ImageJ software (*n* = 3). **F** Endogenous immunoprecipitation was employed to measure the interaction between TLR4 and Trem2. RAW 264.7 cells were stimulated with *Tg*Ag (5 μg/ml) for 24 h, which was followed by immunoprecipitation with Trem2 antibody. TLR4 expression was measured by western blotting (*n* = 3). *Tg*Ag: *T. gondii* antigens, Con: untreated group. Significant changes were determined with a two-tailed unpaired Student *t*-test (**A**–**F**) at *P* < 0.05. *P < 0.05

Subsequently, we performed in vitro experiments to simulate *T. gondii* infection by treating RAW 264.7 cells with *Tg*Ag. To further explore the impact of *Tg*Ag on the downstream TLR4 signaling molecules, both western blotting and cellular immunofluorescence assay were employed to measure the expression of TLR4, P-JNK, TRAF6, and Trem2. *Tg*Ag treatment led to the upregulation of TLR4, P-JNK, and TRAF6 expression while simultaneously downregulating Trem2 expression (Fig. 2A–E). The results observed in the placental tissues were in agreement with these findings, further supporting the involvement of this signaling cascade during *T. gondii* infection. Furthermore, we confirmed the interaction between TLR4 and Trem2 via co-immunoprecipitation and multiplex immunofluorescence, and observed a decrease in the abundance of TLR4-Trem2 complexes upon *Tg*Ag stimulation (Fig. 2F).

**Fig. 3:**
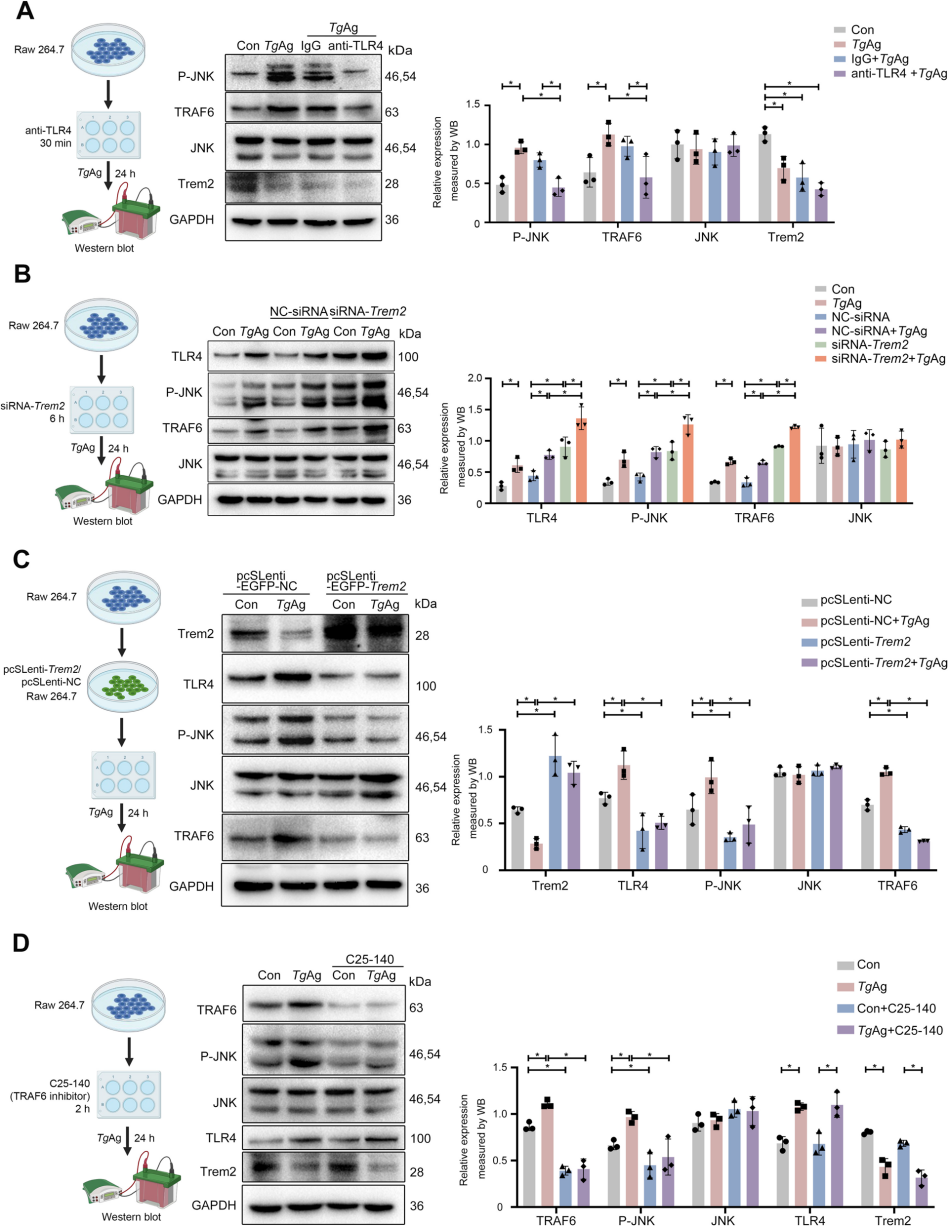
*Toxoplasma gondii* antigens inhibited Trem2 to activate the TLR4/TRAF6/JNK axis. **A** Schematic representation of the treatment of TLR4 blocking antibody on RAW 264.7 cells. The cells, primed with TLR4 blocking antibody (20 μg/ml) or isotype control for 30 min, were stimulated with/without *Tg*Ag (5 μg/ml) for 24 h. Expression of P-JNK, JNK, Trem2, and TRAF6 was assayed by western blotting (*n* = 3). **B** Schematic diagram of *Trem2* knockdown in RAW 264.7 cells. Cells that were transfected with si-*Trem2* or negative control (si-NC) for 6 h were stimulated with/without *Tg*Ag (5 μg/ml) for 24 h. Expression of P-JNK, JNK, TLR4, and TRAF6 was measured by western blotting (*n* = 3). **C** Schematic diagram of *Trem2* overexpression in RAW 264.7 cells. Cells were stimulated with or without *Tg*Ag for an additional 24 h. Expression of P-JNK, JNK, TLR4, Trem2, and TRAF6 was assayed by western blotting (*n* = 3). **D** Schematic illustration of the treatment with the TRAF6 inhibitor on RAW 264.7 cells. Cells, primed with 20 µM C25-140 (TRAF6 inhibitor) for 2 h, were exposed to *Tg*Ag (5 μg/ml) for 24 h. Expression of P-JNK, JNK, TLR4, Trem2, and TRAF6 was assayed by western blotting (*n* = 3). Created in BioRender. Cao, Y. (2025) https://BioRender.com/mwqhkc7. *Tg*Ag: *T. gondii* antigens, Con: untreated group. Significant changes were determined with two-way ANOVA with Sidak’s multiple comparisons test (**A**–**D**) at *P* < 0.05. *P < 0.05

Next, we administered a TLR4-blocking antibody to RAW 264.7 cells, and the results demonstrated that TLR4 blockage effectively attenuated the activation of P-JNK and TRAF6 induced by *Tg*Ag (Fig. 3A). However, the treatment with a TLR4-blocking antibody did not exert any discernible effect on Trem2 expression induced by *Tg*Ag (Fig. 3A). To further confirm the impact of Trem2 on the downstream TLR4-dependent signaling molecules in vitro, the *Trem2* gene in RAW 264.7 cells was knocked down using small interfering RNA (siRNA)-*Trem2* (Fig. 3B). Notably, compared with the control group as well as the NC-siRNA group, *Trem2* knockdown led to upregulation of TLR4, P-JNK, and TRAF6 expression, which was exacerbated by further stimulation with *Tg*Ag (Fig. 3B). However, no statistically significant differences emerged in TLR4, TRAF6, and P-JNK expression between the siRNA-*Trem2* group and either the Con + *Tg*Ag or NC-siRNA+*Tg*Ag group (Fig. 3B). We then transfected RAW 264.7 cells with lentiviral vector *Trem2* to overexpress *Trem2* (Fig. 3C). *Trem2* overexpression led to the downregulation of TLR4, P-JNK, and TRAF6 expression; however, this effect was reversed after exposure of RAW 264.7 cells to *Tg*Ag (Fig. 3C). In addition, TRAF6 inhibitor C25-140 was employed to suppress TRAF6 expression [33]. We found that this inhibitor decreased the expression of P-JNK and TRAF6, with no observable effect on TLR4 or Trem2 level (Fig. 3D). Importantly, *Tg*Ag treatment reversed the inhibitory effects of C25-140 on the TRAF6 and P-JNK (Fig. 3D). In aggregate, these in vitro results suggested that *Tg*Ag suppressed Trem2 expression and activated the downstream TLR4/TRAF6/JNK signaling axis.

### *Trem2* deficiency in macrophages further activated the TLR4/TRAF6/JNK signaling cascade

**Fig. 4:**
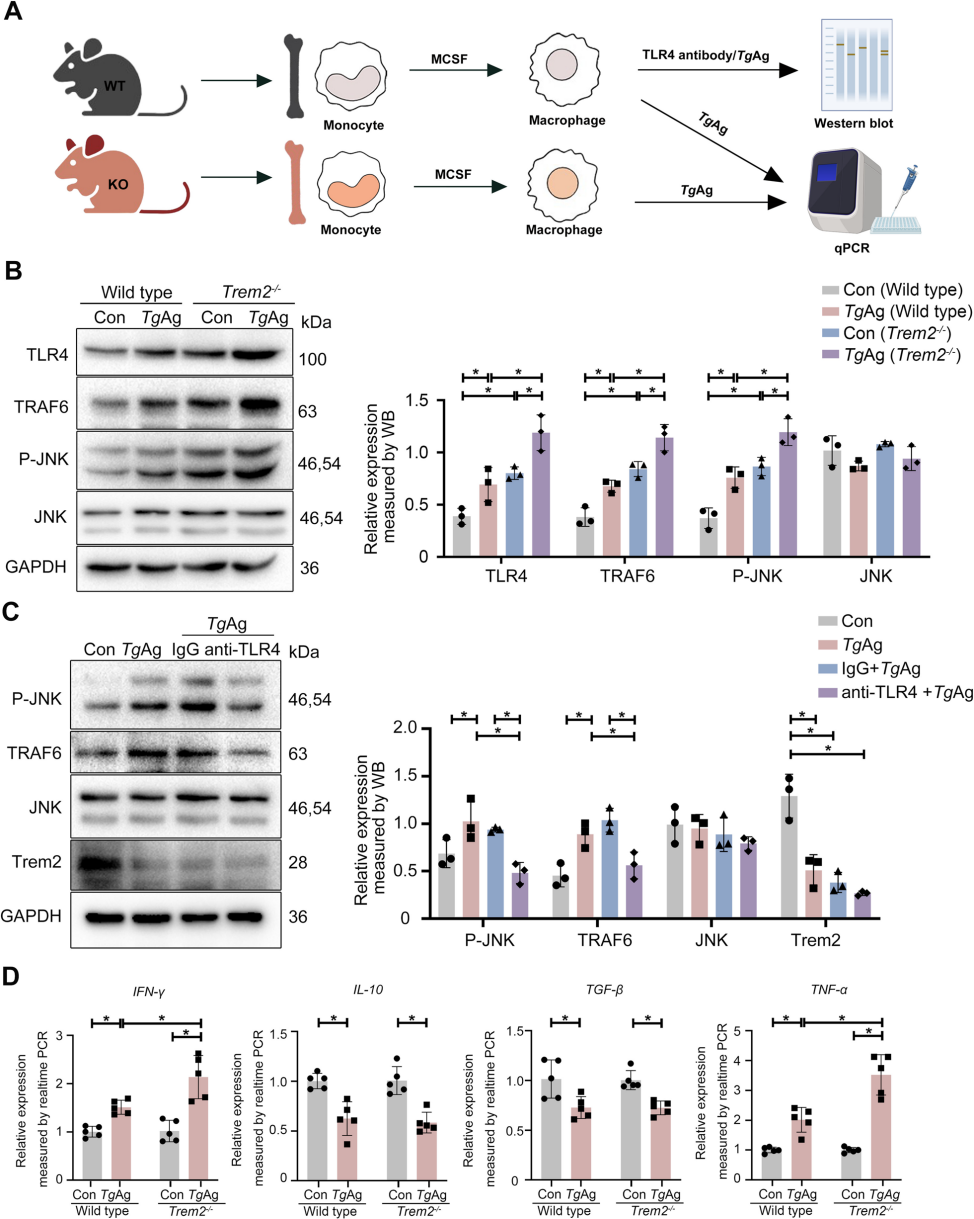
*Trem2* deficiency in macrophages further activated the TLR4/TRAF6/JNK signaling cascade. **A** Flowchart depicting the extraction process of BMDMs derived from WT and *Trem2*^*−/−*^ mice. Created in BioRender. Cao, Y. (2025) https://BioRender.com/mwqhkc7. **B** Expression of P-JNK, JNK, TLR4, and TRAF6 in BMDMs was assayed by western blotting. BMDMs derived from either WT or *Trem2*^*−/−*^ mice were stimulated with/without *Tg*Ag (5 μg/ml) for 24 h (*n* = 3). **C** Expression of P-JNK, JNK, Trem2, and TRAF6 in BMDMs was assayed by western blotting. BMDMs isolated from WT mice, primed with TLR4 blocking antibody (20 μg/ml) or isotype control for 30 min, were stimulated with/without *Tg*Ag (5 μg/ml) for 24 h (*n* = 3). **D** The messenger RNA (mRNA) levels of inflammatory factors in WT or *Trem2*^*−/−*^ mouse BMDMs. BMDMs isolated from either WT or *Trem2*^*−/−*^ mice were exposed to *Tg*Ag (5 μg/ml) for 24 h. Then, the mRNA levels of *TNF-α*, *IL-10*, *IFN-γ*, and *TGF-β* were assayed by real-time PCR (*n* = 5). *Tg*Ag: *T. gondii* antigens, Con: untreated group; anti-TLR4: TLR4/MD-2 Complex Antibody. Significant changes were determined with two-way ANOVA with Sidak’s multiple comparisons test (**B**–**D**) at *P* < 0.05. *P < 0.05

We isolated BMDMs from both *Trem2*^*−/−*^ and WT mice (Fig. 4A). After culturing these cells for 7 days in a complete DMEM medium supplemented with M-CSF, mature BMDMs were obtained [34]. Subsequently, mature BMDMs were stimulated with *Tg*Ag for 24 h in vitro. Experimental data revealed that BMDMs derived from *Trem2*^*−/−*^ mice displayed the elevated levels of TLR4, TRAF6, and P-JNK, in comparison to BMDMs from WT mice (Fig. 4B). In addition, exposure to *Tg*Ag further upregulated the expression of TLR4, TRAF6, and P-JNK in BMDMs derived from *Trem2*^*−/−*^ mice, a pattern that was consistent with findings in the placental tissues from *T. gondii*-challenged mice (Fig. 4B). Additionally, the treatment of BMDMs derived from WT mice with a TLR4 blocking antibody resulted in the downregulation of P-JNK and TRAF6 (Fig. 4C). Subsequently, we examined inflammatory cytokines associated with TLR4 signaling pathway activation [35]. The experimental data demonstrated that *Tg*Ag significantly upregulated *TNF-α* as well as *IFN-γ* in BMDMs derived from WT mice, while *Trem2* knockout in BMDMs further potentiated *Tg*Ag-induced *TNF-α* and *IFN-γ*. Interleukin-10 (*IL-10*) and transforming growth factor beta (*TGF-β*) were downregulated in both WT and *Trem2*^−/−^ BMDMs following *Tg*Ag stimulation. However, *IL-10* and *TGF-β* levels were comparable between *Tg*Ag-treated WT and *Trem2*^−/−^ BMDMs, indicating that *Trem2* deficiency potentiates *TNF-α* and *IFN-γ* transcription. Collectively, these in vitro experiments using BMDMs further substantiated that Trem2 might be a negative regulator of the TLR4-mediated signaling pathway.

## Discussion

Current investigation of Trem2 has largely focused on its activity in microglia, as resident immunocytes in the brain, with particular emphasis on its association with degenerative neurological conditions. However, Trem2 has also been reported to be expressed on human dMφs [36]. Previous studies have indicated that Trem2 expression on dMφs during pregnancy is higher than its expression in peripheral blood [36]. Emerging evidence has indicated a marked decrease in the population of *Trem2*^+^ macrophages in patients with early preeclampsia (EPE), as compared to normal pregnant women [15]. This suggests that Trem2 may function as a beneficial factor helping to maintain normal pregnancy. To confirm the effect of Trem2 in *T. gondii*-induced APOs, we constructed *T. gondii*-infected animal models via intraperitoneal injection. Both oral and intraperitoneal infection routes can elicit host immune responses, though with potential differences in the timing of immune activation. However, there is currently no evidence demonstrating distinct effects of these infection routes on host immunity, particularly maternal–fetal immune tolerance. Notably, intraperitoneal infection remains widely used in studies investigating the mechanisms of *T. gondii*-induced APOs [37]. Hence, in our experimental study, pregnant mice were challenged via intraperitoneal injection with *T. gondii* tachyzoites. We previously found a significant reduction in fetal weight and size of *Trem2*^*−/−*^ pregnant mice challenged with *T. gondii*, in comparison to *T. gondii*-challenged WT mice. Those findings revealed that during *T. gondii* infection, *Trem2* deficiency aggravated APOs in mice [19]. Studies have shown that genetic ablation of *Trem2* enhanced host susceptibility to pathogen infection. It was observed that *Plasmodium berghei*-infected *Trem2*^*−/−*^ mice displayed a markedly higher parasite burden, in comparison to infected WT mice [38]. Similarly, in our previous experiments, an increased parasite burden was also observed in *T. gondii*-infected *Trem2*^*−/−*^ mice, relative to infected WT mice [19]. Therefore, it can be assumed that Trem2 may act as a central regulatory node driving host responses to *T. gondii* infection during pregnancy.

In numerous research models, a complex interaction between Trem2 and TLR4 has been observed. It has been demonstrated that Trem2 can either activate or inhibit the TLR4 signaling cascade. In neuroblastoma, Trem2 interacts with high mobility group box 1 (HMGB1) to form a positive feedback loop, which subsequently activates the TLR4 signaling pathway [39]. However, other studies have suggested negative regulation between Trem2 and TLR4. *Trem2* knockdown has been shown to enhance TLR signaling [29]. In Parkinson’s disease models, *Trem2* knockdown has been linked with the increased expression of TLR4 and MyD88 [20]. Our experimental results supported that genetic ablation of *Trem2* activated the TLR4-dependent signaling pathway. Additionally, *Trem2* deficiency resulted in macrophage hyperresponsiveness to TLR signaling [40]. In the murine osteoarthritis model, *Trem2* deficiency exacerbated M1 macrophage polarization [41]. Since TLR4, a key regulator of M1 polarization, is abundantly expressed in M1 macrophages [42], our data suggested that *Trem2* knockout may induce TLR4 upregulation. Nonetheless, the underlying mechanisms by which *Trem2* knockout activates the TLR4 signaling cascade appear to vary across different cell types. In the CCl4-induced liver injury model in mice, *Trem2* deficiency in Kupffer cells (KCs) exacerbated TLR4-driven inflammation in response to lipopolysaccharide (LPS), by promoting the phosphorylation of ERK and p38 and inhibiting JNK activation [43]. In contrast, *Trem2*-deficient primary microglia treated with LPS or A42-oligomer exhibited more pronounced JNK phosphorylation [44]. The Trem2/DAP12 axis inhibited the inflammation response in microglia through suppressing the activity of the JNK signaling pathway [44]. In addition to DAP12, Trem2 also associates with the transmembrane adapter DAP10 [45]. Interestingly, the DAP10 signaling pathway results in ERK and JNK activation in celiac disease [46]. Hence, the avidity of Trem2-ligand interactions is proposed to determine whether Trem2 exerts activating or inhibitory effects on JNK signaling.

In our experimental model, elevated TLR4 expression was found in the placental tissues from *T. gondii*-infected WT mice. Previous studies have pointed out that TLR4-dependent signaling is pronounced in the pathogenesis of various APOs [47]. For instance, TLR-4, but not TLR-2, was elevated in interstitial trophoblasts from patients with PE, suggesting a potential pathogenic link between TLR-4-mediated innate immune activation and PE [25]. HMGB1, abundantly localized in the placentas from patients with unexplained recurrent spontaneous abortion (URSA), activated pyroptosis via the TLR4/NF-κB pathway to trigger aseptic inflammation, driving the occurrence and progression of URSA [48]. Additionally, it has also been shown that glycosyl-phosphatidylinositol (GPI) derived from *T. gondii* (RH strain) activated TLR4 signaling to participate in an effective host response against *T. gondii* [49]. Administration of a TLR4 antagonist markedly attenuated *T. gondii*-induced ileal inflammation [50], further underscoring the pivotal role of TLR4 signaling during *T. gondii* infection. Notably, single-nucleotide polymorphisms located in the *tlr4* gene can enhance the susceptibility to *T. gondii* and promote the progression of congenital toxoplasmosis in mice [51]. Our research demonstrated elevated TLR4 expression in the placentas from *Trem2* knockout mice following *T. gondii* infection. Hence, pronounced TLR4-dependent signaling might participate in the exacerbated APOs caused by *T. gondii*.

TRAF6 and JNK, as downstream signaling molecules of TLR4, are implicated in the regulation of pregnancy processes [52, 53]. Notably, studies have suggested the elevated TRAF6 expression levels in the decidua from patients with recurrent spontaneous abortion (RSA), consistent with our observation of TRAF6 upregulation in the placentas of infected mice [54]. Furthermore, the dense granule antigen 7 (GRA7) derived from *T. gondii* could promote TRAF6 expression in RAW 264.7 cells, consistent with our in vitro results [55]. In our prior experiments, we demonstrated that *T. gondii* stimulated macrophages to produce inflammatory factors like *IL-12* and *TNF-α* [19]. In line with our observation, Mason et al. found that soluble *Toxoplasma* antigens induced IL-12 secretion in macrophages derived from splenic precursors [56]. Importantly, *TRAF6*^*−/−*^ macrophages, derived from splenic precursors of mice, were treated with soluble *Toxoplasma* antigens, which resulted in the failure of IL-12 production [56]. Additionally, *T. gondii* exosomes could trigger RAW 264.7 cells to produce IL-12 and IFN-γ via JNK activation [57]. Mechanistic investigations revealed that the ROP16I/III effector protein derived from *T. gondii* specifically potentiated transcriptional upregulation of *IFN-γ* [58]. For the establishment of the in vitro model, we employed *Tg*Ag to stimulate RAW 264.7 cells and BMDMs. To assess possible cytotoxic effects mediated by *Tg*Ag, Qiu et al. previously analyzed the antigenic components of *T. gondii* using a quantitative proteomics approach (isobaric tags for relative and absolute quantitation [iTRAQ]–liquid chromatography–tandem mass spectroscopy [LC–MS/MS]), with the data published in *Scientific Reports* (2016) [28]. The analysis identified 3699 *T. gondii* peptides, including 3578 unique peptides corresponding to 984 *T. gondii* proteins. In our previous experiments, treatment with *Tg*Ag alone did not significantly affect trophoblast cell migration, invasion, or proliferation compared to the control group [19]. Therefore, at the concentration used, *Tg*Ag demonstrated no evidence of cellular toxicity. We found that *Tg*Ag stimulation in BMDMs significantly induced high-level expression of the inflammatory cytokine *IFN-γ*, whereas *Trem2* knockout further promoted the upregulation of *IFN-γ*. T*.* gondii in vitro stimulated human dMφs to upregulate P-JNK expression [11]. Taken together, these findings are consistent with our experimental results, indicating that the TRAF6/JNK signaling pathway might contribute to APOs triggered by *T. gondii* infection. Furthermore, our study revealed that *Tg*Ag stimulation in BMDMs induced robust transcription of the inflammatory cytokines like *IFN-γ* and *TNF-α*, but merely triggered low-level transcription of *TGF-β* and *IL-10*. This cytokine expression pattern differed markedly from observations in the BeWo cell model, where *T. gondii* infection activated the TLR4 signaling pathway, resulting in low-level expression of* IL-10*, *TGF-β*, and *TNF-α* [35]. Notably, the study demonstrated that MIC3, a microparticle protein secreted by *T. gondii*, not only induced *TNF-α* secretion in macrophages but also significantly promoted M1-type macrophage polarization [59]. These findings suggest that *T. gondii* may govern cytokine expression profiles through distinct molecular mechanisms across different host cell models.

## Conclusions

We have identified a Trem2-mediated signaling pathway contributing to *T. gondii*-induced APOs. Trem2 was found to interact with TLR4, and the knockout of *Trem2* in mice further promoted the activation of the TLR4/TRAF6/JNK signaling pathway, contributing to more severe APOs. *Trem2* overexpression in macrophages inhibited the TLR4/TRAF6/JNK signaling pathway, whereas *Trem2* knockout in macrophages promoted its activation. *Trem2* loss in macrophages further potentiated *T. gondii* antigen-induced *TNF-α* and *IFN-γ*. We therefore propose that therapeutic strategies promoting Trem2 expression to inhibit the TLR4/TRAF6/JNK signaling pathway could be developed to treat *T. gondii*-triggered APOs.

## Supplementary Information

Additional file 1: Table S1. All the primers for real-time PCR.

## Data Availability

The online version contains supplementary material available at 10.17632/2fyxmnd8b7.2.

## Abbreviations

APOs: Adverse pregnancy outcomes
BMDMs: Bone marrow-derived macrophages
dMφs: Decidual macrophages
ERK: Extracellular regulated protein kinases
GRA7: Granule antigen 7
GPI: Glycosyl-phosphatidylinositol
GAI-9: Galectin-9
HSP70: Heat shock protein 70
HMGB1: High mobility group box 1
IFN-γ: Interferon-γ
IL-10: Interleukin-10
KCs: Kupffer cells
MAPK: Mitogen-activated protein kinase
MMPs: Matrix metalloproteinases
MyD88: Myeloid differentiation primary response protein 88
ROS: Reactive oxygen species
SDS–PAGE: Sodium dodecyl sulphate–polyacrylamide gel electrophoresis
TAB2: TAK1-binding protein 2
TGF-β: Transforming growth factor beta
*T. gondii*: *Toxoplasma gondii*
TLR4: Toll-like receptor 4
TRAF6: TNF receptor-associated factor 6
Trem2: Triggering receptor expressed on myeloid cells 2
TNF-α: Tumor necrosis factor alpha
WT: Wild type
