# Supplementary material for: Toxoplasma gondii-induced adverse pregnancy outcomes: insight into the inhibitory role of Trem2 on TLR4/TRAF6/JNK signaling pathway
Source: Parasit Vectors. 2025 Oct 6;18:396. doi: 10.1186/s13071-025-07000-w (PMC12502315; doi:10.1186/s13071-025-07000-w)
Supplement: Supplementary file 1 — Additional file 1: Table S1. All the primers for real-time PCR. [file 13071_2025_7000_MOESM1_ESM.docx]

Additional file 1: Table S1.

Table S1. All the primers for real-time PCR.

| Gene name | Primer name | Primer sequences (5'-3') |
| --- | --- | --- |
| *GAPDH* | *GAPDH*-F | TGGAAAGCTGTGGCGTGAT |
|  | *GAPDH*-R | TGCTTCACCACCTTCTTGAT |
| *IL-10* | *IL-10*-F | GCTCTTACTGACTGGCATGAG |
|  | *IL-10-*R | CGCAGCTCTAGGAGCATGTG |
| *IFN-γ* | *IFN-γ*-F | ATGAACGCTACACACTGCATC |
|  | *IFN-γ*-R | CCATCCTTTTGCCAGTTCCTC |
| *TGF-β* | *TGF-β*-F | CGGTGCTCGCTTTGTA |
|  | *TGF-β*-R | GCCACTCAGGCGTATC |
| *TNF-α* | TNF-α-F | AGGCACTCCCCCAAAAGATG |
|  | TNF-α-R | CCACTTGGTGGTTTGTGAGTG |
